# Defective Mitochondrial Function *In Vivo* in Skeletal Muscle in Adults with Down’s Syndrome: A ^31^P-MRS Study

**DOI:** 10.1371/journal.pone.0084031

**Published:** 2013-12-31

**Authors:** Alexander C. Phillips, Alison Sleigh, Catherine J. McAllister, Soren Brage, T. Adrian Carpenter, Graham J. Kemp, Anthony J. Holland

**Affiliations:** 1 Department of Psychiatry, University of Cambridge, Cambridge, United Kingdom; 2 Wolfson Brain Imaging Centre, University of Cambridge, Cambridge, United Kingdom; 3 MRC Epidemiology Unit, Institute of Metabolic Science, University of Cambridge, Cambridge, United Kingdom; 4 Department of Musculoskeletal Biology, University of Liverpool, Liverpool, United Kingdom; Mayo Clinic, United States of America

## Abstract

Down’s syndrome (DS) is a developmental disorder associated with intellectual disability (ID). We have previously shown that people with DS engage in very low levels of exercise compared to people with ID not due to DS. Many aspects of the DS phenotype, such as dementia, low activity levels and poor muscle tone, are shared with disorders of mitochondrial origin, and mitochondrial dysfunction has been demonstrated in cultured DS tissue. We undertook a phosphorus magnetic resonance spectroscopy (^31^P-MRS) study in the quadriceps muscle of 14 people with DS and 11 non-DS ID controls to investigate the post-exercise resynthesis kinetics of phosphocreatine (PCr), which relies on mitochondrial respiratory function and yields a measure of muscle mitochondrial function *in vivo*. We found that the PCr recovery rate constant was significantly decreased in adults with DS compared to non-DS ID controls (1.7±0.1 min^−1^ vs 2.1±0.1 min^−1^ respectively) who were matched for physical activity levels, indicating that muscle mitochondrial function *in vivo* is impaired in DS. This is the first study to investigate mitochondrial function *in vivo* in DS using ^31^P-MRS. Our study is consistent with previous *in vitro* studies, supporting a theory of a global mitochondrial defect in DS.

## Introduction

Down’s syndrome (DS) is a developmental disorder associated with triplication of chromosome 21, and affects 1 in 700–1000 live births [1], [2]. The clinical phenotype in DS is variable and multi-faceted and includes specific physical characteristics and an increased risk for developing sensory deficits, congenital heart defects, early onset Alzheimer’s disease (AD), and hypothyroidism [3]. DS is also associated with obesity and reduced exercise capacity [4]. We have recently reported reduced physical activity levels in DS compared to people with intellectual disability (ID) not due to DS, with the older people with DS being less active than their younger counterparts [5]. As this use of an ID control group eliminates a number of general (social and environmental) factors that might explain this observation, we postulated an underlying age-related pathophysiology which limits activity in people with DS, an obvious candidate for which is mitochondrial dysfunction [5].

Mitochondrial generation of adenosine triphosphate (ATP) is fundamental to cellular energy turnover, and defects of mitochondrial function manifest in a variety of ways in specific clinical syndromes and in chronic diseases. In general defective mitochondrial function *in vivo* results in limited capacity for ATP synthesis, and may be associated with increased intracellular levels of toxic reactive oxygen species (ROS). *In vitro* studies in DS have demonstrated mitochondrial abnormalities including mtDNA mutations [6], global dysregulation of genes associated with mitochondrial function in the foetus [7], and mitochondrial enzyme deficiency [8]. However, we are aware of no studies that have assessed mitochondrial function *in vivo* in DS. We therefore undertook a study of DS mitochondrial function *in vivo* in skeletal muscle using ^31^P magnetic resonance spectroscopy (^31^P-MRS), which offers a non-invasive method for measuring mitochondrial function *in vivo* by analysing the post-exercise recovery kinetics of phosphocreatine (PCr), a process which relies on mitochondrial production of ATP [9]. We postulated that participants with DS would demonstrate a slowed recovery of PCr, indicating a defect of mitochondrial function *in vivo*, compared to control participants with similar levels of ID but not due to DS.

## Methods

### Ethics Statement

Ethical approval for the study was granted by the Cambridgeshire 3 Research Ethics Committee and all studies were conducted in accordance with the principles of the Declaration of Helsinki. Only participants judged to have the capacity to consent in accordance with the Mental Capacity Act (2005) were recruited. Written consent was obtained from all participants.

### Recruitment, Screening and Familiarisation

Participants with ID not due to DS were deliberately chosen as controls for the DS group in an attempt to match for level of intellectual disability, environmental factors and fitness levels. Participants were recruited from a database of participants in a previous study [5] and were initially selected to match as far as possible age, gender, BMI and physical activity levels between groups. We scanned 29 individuals, but after detailed consideration when matching activity levels of the two groups, selected for analysis 14 DS and 11 ID participants. Inclusion criteria were: mild to moderate ID; known to ID services; aged ≥12 y; able to walk unaided; and able to understand protocol instructions. Exclusion criteria were: smoking; autism spectrum disorder; diabetes mellitus, cardiovascular disease; medication known to effect energy metabolism; and standard magnet contraindications. Prior to inclusion, participants underwent a preliminary screening at their home residence to determine whether they would be able to understand and complete the study protocol, and to assess their capacity to consent. Two weeks prior to the experimental measurements participants were shown a video and underwent at least two familiarisation sessions at their home residence to ensure they were comfortable with the in-scanner exercise protocol. All participants underwent at least four separate practice trials.

### Measurements of Height, Weight, and Body Fat

Weight was measured to the nearest 0.1 kg with the participant dressed in lightweight clothing and without shoes, using a calibrated electronic scale (Seca 813, Seca Ltd, UK). Height was measured by the stretch stature method to the nearest 0.5 cm using a portable stadiometer (Seca Leicester Height Measure, Invicta Plastics, UK). Percentage body fat was assessed by bioelectrical impedance using a TBF−521 analyser (Tanita Corp., Japan) in accordance with the manufacturer's instructions.

### Measurement of Leg Strength

Two weeks prior to the experimental measurements isometric leg strength was measured at the participants’ home residence using a portable back and leg dynamometer (Takei, model T.K.K. 5162, Japan) according to a protocol [10] which has been validated in both adolescents and adults [10], [11].

### Assessment of Total Physical Activity

Habitual physical activity was assessed using an Actigraph GT1M accelerometer (Actigraph LLC, Pensacola, FL, USA) for seven consecutive days. Accelerometers have been used previously to assess physical activity in a number of studies in DS [12]–[14]. The total physical activity measure was used as a measure of habitual physical activity in these participants and not used to make inferences on energy expenditure [14]. These participants formed part of a previous report on activity in these cohorts and the methodology and analysis were as previously described in that study [5].

### ^31^P-MRS Measurement of Post-exercise PCr Recovery Kinetics

^31^P-MRS data were acquired on a Siemens MAGNETOM 3T Verio scanner. Participants were asked to arrive having refrained from strenuous exercise for at least 24 h and having abstained from alcohol, caffeine, and food for the preceding 24 h, 6 h, and 2 h respectively. The volunteers were placed supine and a 9 cm diameter surface coil attached to the right quadriceps (1/3 distal). An MR-compatible weight was attached to the right ankle that was proportional to their previously-determined leg strength, such that the PCr concentration after exercise was targeted to fall by 20–30% of basal levels, thereby avoiding any significant lowering of pH which is known to complicate interpretation of PCr recovery kinetics. The exercise paradigm consisted of 1 min rest, 1 min knee extensions (0.5 Hz), then 4 min rest. This was then repeated to permit two measurements of post-exercise PCr recovery, results from the analysis of which were averaged. Spectra were acquired with a TR of 2s and as previously described [15]. The PCr recovery rate constant, k, was found using a two parameter monoexponential fit to the equation: PCr(t) = PCr_initial_+(PCr_end_ – PCr_initial_)(1-exp(-k.t)), where t is the time from the start of recovery, PCr_initial_ and PCr_end_ are the PCr content at the initial and end of recovery phases respectively. MATLAB software (The MathWorks, Natick, MA, USA) was used to calculate PCr_end_ from the mean end-recovery PCr values and then to obtain k and PCr_initial_ by the Levenberg Marquardt fitting algorithm. All spectra were analysed in jMRUI [16] and fitted using the AMARES [17] algorithm with prior knowledge. The intracellular pH was determined from the chemical shift of inorganic phosphate relative to PCr. A fully relaxed spectrum with 8 averages was also obtained for the calculation of metabolite ratios and concentrations. The conventional assumption was made that the intracellular concentration of ATP was 8.2 mM, and the free concentration of adenosine diphosphate (ADP) was calculated using established methods [18] with the conventional assumption (in the absence of any reported data in DS) of total creatine = 42.5 mM. The signal-to-noise ratio (SNR) of the PCr recovery sequence was defined as the (mean signal)/SD of the recovered PCr values (20 points, TR = 2s). High subcutaneous adipose tissue thicknesses can result in a low SNR and uncertainty in the accuracy of the PCr recovery rate constant, and hence a cut off value of SNR <35 was chosen for exclusion of k values.

### Statistics

Statistical analysis was performed in IBM SPSS Statistics 21 (IBM Inc., Armonk, NY, USA), with significance set at *p*<0.05. A two-tailed independent samples t-test was performed to detect significant differences between groups.

## Results

Participants with ID not due to DS and participants with DS were matched for age, gender, BMI and physical activity levels, and were found to have similar percentage body fat (Table 1). However, the DS were significantly shorter than the ID controls (*p = *0.001). No significant correlation of total physical activity with age was found in either group, or altogether.

**Table 1:** Characteristics of the participants with Down’s syndrome (DS) and those with a non Down’s syndrome related intellectual disability (ID).

	DS (n = 14)	ID (n = 11)	*p*-value
Age (yrs)	32.7 (2.2)	29.8 (2.7)	0.410
Gender	8 M, 6 F	5 M, 6F	0.580
Weight (kg)	72.2 (3.2)	80.0 (4.9)	0.175
Height (m)	1.55 (0.03)	1.72 (0.04)	**0.001**
BMI (kg/m^2^)	30.4 (1.7)	26.9 (1.1)	0.100
Percentage body fat (%)	27.5 (2.7)	27.5 (3.1)	0.991

Data presented are mean (SEM).

M, Male; F, Female; BMI, body mass index.

**Figure 1:**
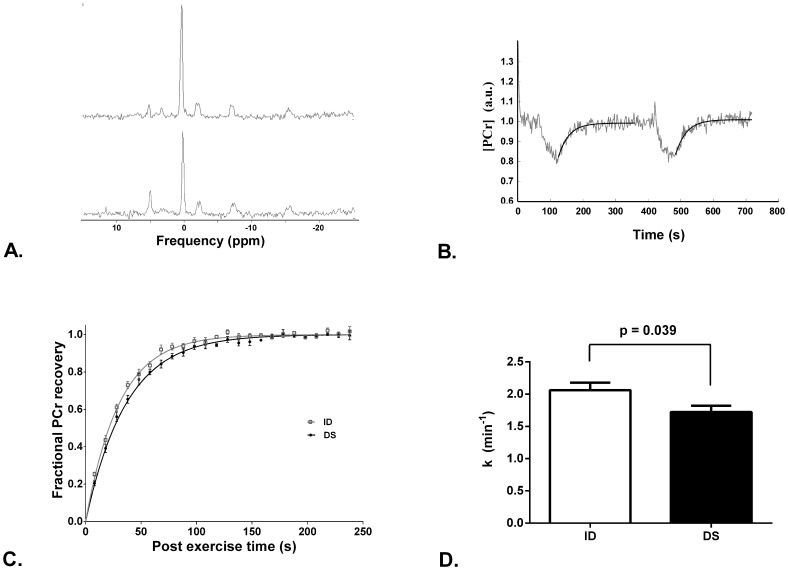
^31^P-MRS measures of mitochondrial function. (A) Representative single spectra (NA = 1) at rest (upper) and near end exercise (lower), with 2 Hz line broadening applied for illustrative purposes. (B) Corresponding PCr timecourse to the spectra in A, which illustrates the typical [PCr] end-exercise depletion in this study. The SNR of this timecourse was 46, significantly below average. The black line represents the monoexponential fit to both PCr recoveries. The recovery time constant, k, was 1.87 min^−1^ and 1.83 min^−1^ for recovery from exercise bouts 1 and 2 respectively. (C) Mean fractional PCr recovery curves for physical activity matched ID (grey squares; n = 11) and DS (black circles; n = 10). Five spectra were averaged to give a time resolution of 10 seconds for clarity in this figure. The monoexponential fit using the mean recovery rate constant is shown for ID (grey line) and DS (black line). (D) Recovery rate constant (k) for matched physical activity groups of ID (white bars; n = 11) and for DS (black bars; n = 10). In C–D, data are mean +/− SEM.

Four participants with DS were eliminated from the calculation of k due to not meeting the SNR requirements described in the Methods section, and hence the ^31^P-MRS and accelerometry results in Table 2 represent the DS group with n = 10. Post-exercise PCr recovery was significantly slowed in the DS group compared with ID (*p = *0.039, Figure 1). Neither the resting nor end-exercise intracellular pH (pH_i_) differed significantly between those with DS and those with ID without DS (Table 2), and end-exercise pH_i_ was not significantly lower than resting pH_i_ in either group. No differences in resting metabolite concentrations nor their ratios were found between the groups (Table 2). There was no significant correlation between total physical activity and the PCr recovery rate constant, k in either group, or altogether.

**Table 2:** Accelerometry and ^31^P-MRS measurements for the participants with Down’s syndrome (DS) and those with a non Down’s syndrome related intellectual disability (ID).

Category	Subcategory	DS (n = 10)	ID (n = 11)	*p*-value
Accelerometry	Total physical activity (counts/min)	542.5 (25.3)	611.6 (35.1)	0.133
^31^P-MRS	Exercise weight (kg)	2.03 (0.10)	2.77 (0.29)	**0.031**
	k (min^−1^)	1.72 (0.10)	2.06 (0.12)	**0.039**
	Resting Pi:PCr	0.086 (0.005)	0.096 (0.006)	0.216
	Resting PDE:PCr	0.123 (0.011)	0.115 (0.007)	0.567
	Resting Pi:PDE	0.73 (0.06)	0.86 (0.07)	0.213
	Resting [Pi] (mM)	2.85 (0.16)	2.96 (0.17)	0.633
	Resting [PDE] (mM)	4.07 (0.35)	3.58 (0.22)	0.244
	Resting [PCr] (mM)	33.4 (0.7)	31.2 (0.9)	0.073
	End exercise [PCr] (mM)	26.5 (1.5)	24.3 (0.9)	0.211
	Resting [ADP] (µM)	14.7 (1.8)	20.2 (2.3)	0.078
	End exercise [ADP] (µM)	36.8 (4.9)	45.2 (4.6)	0.227
	Resting pH_i_	7.017 (0.015)	7.036 (0.007)	0.281
	End exercise pH_i_	7.061 (0.010)	7.068 (0.009)	0.648

Data presented are mean (SEM).

k, PCr recovery rate constant; [Pi], concentration of inorganic phosphate; [PCr], concentration of phosphocreatine; [PDE], concentration of phosphodiester; [ADP], concentration of adenosine diphosphate; pH_i_, intracellular pH.

## Discussion

We found significantly slower post-exercise PCr recovery in skeletal muscle of the participants with DS compared with the non-DS ID control participants. Post-exercise PCr recovery, conveniently measured as the recovery rate constant, which was 16% lower in the DS participants, is a measure of mitochondrial function *in vivo* which integrates the contributions of muscle mitochondrial numbers, the mitochondrial content of enzymes, transporters and respiratory chain complexes, the activity of relevant cytosolic enzymes, and the whole-body cardiorespiratory physiology affecting substrate and oxygen supply to active muscle [19]. In exercise of the kind used here the exercising muscle mass is very small (compared e.g. to bicycle ergometry [20]), and cardiovascular limitations to this measure are normally negligible. Our participants with DS had no clinical evidence of cardiovascular or pulmonary disease, and therefore no reason to suppose that the defect we identified lies ‘upstream’ of the mitochondrion. The likeliest explanation is either a lower mitochondrial content, or a defect in intrinsic mitochondrial metabolism.

There are various lines of *in vitro* evidence to support, or at least parallel, our findings of mitochondrial dysfunction *in vivo* in DS. Previous *in vitro* studies in DS have shown mtDNA mutations or mitochondrial dysfunction in brain [6], [21]–[23] and dysregulation of genes associated with mitochondrial function in foetal heart tissue [7]. More specifically lower mRNA levels of complex I and decreased protein levels in complex I subunits [21] as well as decreased levels of complex V [24] have been identified in the brains of people with DS. Levels of mitochondrial enzymes in platelets, including cytochrome oxidase (COX), have been shown to be significantly lower in people with DS compared with controls [8]. There is also evidence of compensation for mitochondrial inefficiency, including a 25% increase in mitochondrial mass of fibroblasts in people with DS [25]. A recent study by Helguera et.al. [26] has shown that mitochondria move more slowly in DS neurons compared with controls, although there were higher numbers of moving mitochondria in DS at any given time that may be a compensatory mechanism. Collectively the results from these studies suggest a systemic long standing dysfunction of mitochondria in DS. To our knowledge, there have been no such studies in DS investigating skeletal muscle, which for practical reasons is the only tissue in which mitochondrial function *in vivo* (i.e. the capacity for mitochondrial ATP synthesis) can be quantified non-invasively [19]. The results of our *in vivo* study in skeletal muscle support this theory of a global mitochondrial defect.

We have previously shown that people with DS have highly sedentary lifestyles compared to their non-DS ID peers [5]. Similarly low levels of habitual physical activity have recently been reported in people with mitochondrial disease [27]. In addition, the authors of that study found a high prevalence of obesity, and a moderate negative correlation between steps walked per day and disease severity. We suggest that low levels of activity, the reduced capacity for exercise [4] and high rates of obesity [28] in DS could be accounted for in part by reduced mitochondrial ability of skeletal muscle to generate ATP.

However, any cross-sectional study relating habitual activity to muscle energetics (in this case, muscle mitochondrial capacity) must address the issue of causal direction; whilst mitochondrial dysfunction *in vivo* can certainly contribute to reduced habitual activity, it is also possible that reduced activity may lead to impaired muscle mitochondrial function. In the present study we have tried to address this by matching the DS and non-DS ID groups for physical activity. The data does not exist to say how much a ‘primary’ reduction in habitual activity would be necessary to yield the 16% defect we observed in mitochondrial function *in vivo* in DS compared to non-DS ID, nor conversely how much a primary mitochondrial defect (in mitochondrial numbers or intrinsic mitochondrial activity) of this size would be expected, on its own, to limit activity. We do know that the ‘slope’ of the relationship between these two measures is steep, in the sense that symptomatic primary mitochondrial myopathy is associated with on average a 40–50% defect in skeletal muscle mitochondrial capacity as measured by ^31^P-MRS [29]. The defect we have identified here is therefore likely to be functionally significant, even if we cannot be sure whether it is cause or effect (or both).

Precocious ageing and Alzheimer’s disease (AD) are strikingly common in DS, with almost 100% of people with DS eventually developing the neuropathology of AD [30], which is likely to be linked to the overexpression of the amyloid precursor protein gene (*APP*) resulting in a lifelong excess of beta-amyloid (Aβ). The role of mitochondrial dysfunction in neurodegenerative diseases is well-established [31]. Aβ has been shown to be imported into mitochondria where it inhibits function [32], [33], and data indicate that mitochondrial dysfunction contributes to the development of DSAD [34]. Aβ deposits have been seen in non-brain tissue of people with AD, DS and in normal ageing [35]. Further, transfer of the *βAPP* gene into cultured normal human muscle leads to decreased mitochondrial enzyme activity and structural abnormalities [36]. This demonstrates that mitochondrial interaction with Aβ is not limited to brain, and suggests, further, that mitochondrial dysfunction quantified in muscle *in vivo*, as in the current study, may provide insight into Aβ and mitochondria in brain. Mitochondrial dysfunction and mutations of mtDNA are a major hallmark of human ageing [37], suggesting that mitochondria may underlie the precocious biological ageing that is characteristic of DS. In the current study, there was no significant correlation with age in the DS group (*p* = 0.165, data not shown) although the eldest person in the group was only 44 y.

Low activity levels in DS are associated with reduced life expectancy, irrespective of heart disease [38]. Increasing mobility of people with DS should therefore improve well-being as well as mortality. Furthermore, high levels of exercise in people over 65 y in the general population has been shown to be protective against cognitive decline [39], adding further support to the notion that reduced habitual activity in DS may impact on the rate and severity of dementia, although mitochondrial dysfunction may underlie both these aspects of the phenotype. Understanding reasons for reduced habitual activity in DS, including the role of mitochondrial dysfunction and accumulation of mtDNA mutations, may be key to a number of health issues associated with the syndrome.
